# The Impact of Physiologic and Non-Physiologic Delivery on the Mother and Neonate Outcomes; A Comparative Study on the Primi Gravid Mothers

**Published:** 2015-03

**Authors:** Maryam Khooshide, Tiba Mirzarahimi, Ghodrat Akhavan Akbari

**Affiliations:** 1 Department of Obstetrics and Gynecology, Arash Hospital, Tehran University of Medical Sciences, Tehran, Iran; 2 Department of Anesthesiology Fatemi Hospital, Ardebil University of Medical Sciences, Ardebil, Iran

**Keywords:** Physiologic labor, facilitated labor, mother outcomes, neonate outcomes

## Abstract

**Objective:** To compare the effect of the physiologic and facilitated labor on the mother and neonate outcomes in the prim gravid women referring to Arash Hospital.

**Materials and methods:** This clinical trial study was performed on 200 low risk pregnant women referring to Arash Women’s Hospital in 2012-2013. Mothers were divided into two groups of 100 patients using a simple random sampling method. The first group received the on-pregnancy and physiologic labor training and the second group was nominated for facilitated labor without training. The mother and neonate outcomes in these two delivery methods were then compared.

**Results:** The rate of cesarean section in the physiologic group was significantly lower compared with the intervention group (p = 0.001). Also in the first stage of labor, VAS was measured to be noticeably lower in the physiologic group in comparison with the intervention group (p = 0.001), while the difference of VAS between the two studied groups was found not to be significant in the second stage of labor. In terms of duration of the labor and neonatal Apgar score two groups were not considerably different (p > 0.05). Moreover, the laceration rate in the physiologic group was determined to be noticeably higher as compared to the intervention group (p = 0.001). The groups were considerably different in terms of the vaginal bleeding and maternal satisfaction (p = 0.001).

**Conclusion:** This study revealed the lower rate of cesarean section, abnormal vaginal bleeding and pain score in the physiologic group compared with the facilitated group. Moreover, mothers of the first group were more content with the labor process.

## Introduction

Labor is a natural phenomenon. On one hand, medical improvements have made a great progress in safety of the delivery; on the other hand, current medical interventions in the labor process, have led to a rise in the rates of cesarean sections. These days, the cesarean section rate has noticeably increased and its unnecessary cases are concerning modern obstetrics. Cesarean not only causes the maternal morbidity and mortality but it also threatens the neonate health. Cesarean section causes the neonate death 2-3 times more than the vaginal delivery (1). Babies born through cesarean are 5 times more in the risk of initial pulmonary blood pressure compared with the babies born through vaginal delivery (2). However, cesarean section is inevitable when there is scientific indication. Since 1960, applying Active Management of Labor (AMOL) protocol has been dramatically improved. It is claimed that active management protocol has led to the great mother and neonate outcomes and also a decrease around 18% in the cesarean section rates. The main aim of this protocol is to shorten the duration of the labor process. Infection, physical-neurological disorders, neonate death, abnormal vaginal bleeding, post-labor infection, mental disruption, insomnia and exhaustion in mothers are considered as the results of long labor time (1). Based on this protocol, the cervical dilation of patients when admitted is measured to be 2-3 cm. But in the case of definite childbirth diagnosis at the time of admission, the patient undergoes amniotomy procedure, no matter how long the cervical dilation is. Diagnosis of the labor is the key factor in AMOL protocol. In the case of incorrect diagnosis, mother receives the induced labor which in turn augments the risk of the cesarean section (3). According to the previous reports, active management protocol has made the labor rate increase of 98% in only 12 hours. Also the frequency of cerebral palsy was 2 out of 1000 births and AMOL consequent childbirth trauma has been scarcely reported. In active management, epidural analgesia, showing no significant effect on the cesarean rate could also be used. It has been found when active management is applied; because of the dystocia, only 10% of pregnant women undergo cesarean section. Another study revealed that the rate of cesarean decreased to 34% due to the dystocia. Contrary to these findings, one report indicated that AMOL protocol has not made a decrease in cesarean rates, although another study showed a decrease of 25-50% in the cesarean rate. According to the World Health Organization principles the main aim of the vaginal delivery is to provide the best conditions for the mother and infant. Based on the statements of this organization, as the first intervention leads to a cascade of interventions which might cause disruption in the physiologic labor course and threaten the mother and neonate health, there should be a sensible reason for any kinds of intervention in natural vaginal delivery. The principles of this course determined by WHO includes individual care and assistance, regular fetal heart rate (FHR) and fetal movements control, the least intervention and also vaginal examination (4, 5). The physiologic labor is described to be safe for mother, natural, with low pain and no medical interventions in a an anxiety free atmosphere in which mother`s hormonal system modulates the main labor factors. This hormonal function would be disrupted by the induction, taking painkillers, preventing mother from moving and the separation of mother and the infant. A wide variety of interventions could be carried out during the delivery, all aiming to shorten the duration of the labor process of which the oxytocin induction is of the great importance. Based on a study performed in New York in 2006, the oxytocin induction enhances the risk of the neonatal respiratory distress (6). According to the findings of this study and other similar ones, WHO has stated that 10% of the deliveries are allowed to be induced and intensifying mother’s labor pain is permissible in specific cases (4). Regarding this principle, some studies indicate that the selective induction increases the need for anesthesia, epidural analgesia and neonatal resuscitation, leading to an increase in the cesarean rates. Also applying tools, fever, dystocia, low birth weight and long-term hospitalization were all considered to be associated with the selective induction (7). Based on a study performed in the U.S, some medical-labor interventions have been commonly used including the regular FHR and venous fluid control, epidural and spinal anesthesia, vaginal examination, applying urine probe, rupturing the membrane and using oxytocin. The number of mothers receiving these 7 interventions is daily increasing without any findings based on the extensive studies. The constant FHR control and vaginal examinations cause a decrease in mothers’ body movements. Therefore, mother is not able to change her position in the response to pain. This results in receiving more interventions such as pain killers (8).In addition, the post-labor infection has been found to be less in the physiologic group compared to the intervention group (9). Another running intervention is episiotomy. Some studies have shown that not only this procedure does not benefit the mother and neonate but it also could result in fecal incontinence after delivery (10). According to these consequences, WHO has limited the usage of episiotomy to 10% of the certain cases (5, 9). This study was carried out to compare the physiologic and facilitated delivery and to investigate the effects of these two delivery ways on the mother and neonate outcomes in pregnant women referring to Arash Women’s Hospital in 2012-2013.

## Materials and methods

This clinical trial study was performed on 200 prim gravid mothers referring to Arash Women’s Hospital for the prenatal and labor care. This study was approved by the ethics committee and the informed consent was obtained from all the participants. Then they were divided randomly into two equal groups. The first group received the on-pregnancy training and physiologic labor routine care. The second group underwent the on-pregnancy and facilitated labor routine care without training.

Mothers included in this study were supposed to be 18-35 years old, prim gravid, singleton pregnant, at the 37^th^ – 40^th^ week of gestation, with normal Body Mass Index of 23-30. They were also expected not to have any bad medical or obstetrics and drug consumption history with tendency to undergo natural vaginal delivery.

Cases with hypertension, diabetes, vaginal bleeding, preterm childbirth, membrane rupture of the amniotic sac 24 hours prior to onset of the labor and mothers carrying macrosom fetus were excluded from our study.

Since the 20^th ^-37^th^ week of gestation, mothers attended 8 training sessions (each session lasted 90 minutes) to get informed about the labor process, methods to relieve the delivery pain such as »SELF HELP« technique, physiological changes, on-pregnancy nutritional care, and lactation period. These courses were also held with the aim of enlightening mothers’ family members especially their spouses about infant health care and emotional support for mothers. The on-labor training consisted of the breathing techniques, relaxation, perineal massage techniques, correct labor position and skeletal muscular exercise. Furthermore, mothers were told to avoid (1) being fast before the labor, (2) angio catheter IV lining, (3) limiting their movements during the labor process (4) receiving litotomy, induction or episiotomy (unless needed). Trained mothers were candidates for the non-intervened physiologic childbirth and hospitalized in their active labor phase with the dilation of 4 cm. In the first stage of labor, the vaginal examinations and FHR control were done every 120 minutes and every 30 minutes respectively, whereas in the second stage these times decreased to every 30 minutes for the vaginal examinations and 15 minutes for FHR control. During the labor process, mothers were permitted to drink liquids within limits. In order to keep IV catheters open, Heparin Lock was applied. A trained midwife (doula) assisted the patients during the process. In the case of failure to progress in the delivery such as the cervical dilation lower than 1.2 cm/h and fetal descent lower than 1 cm/h, Cephalopelvic) disproportion diagnosis was conducted. If the patient was not diagnosed with CPD, the labor augmentation and induction based on the following protocol were carried out. A 1-ml ampoule containing 10 units of oxytocin was diluted into 100 ml of the lactated ringer solution by an infusion pump. The starting dosage was 4 mu/min and if needed the dosage increased to 6 mu/min every 15 minutes (with the maximum dosage of 64 mu/min). The physiologic labor cases did not receive the episiotomy as a routine fact but in the case of macrosomia or hard perineum, regarding mothers’ demands, episiotomy was performed. Also the pain intensity in some willing cases caused applying pushing method. The second group of mothers in the latent or active phase of labor was hospitalized and controlled hourly in terms of IV lining, augmentation, monitoring and vaginal examinations. The amniotomy, Oxytocin induction based on the mentioned protocol and episiotomy were performed for this group. During the labor process, the intensity of mothers’ pain was measured every 30 minutes using VAS. This scale consisted of the numbers from 0 to 10 (0-3 for the mild pain, 4-6 for the moderate pain, 7-9 for the severe pain and >10 for the intolerable pain). The average pain score throughout the first and the second stage of labor, type of labor, amount of vaginal bleeding after childbirth, the duration of labor process, infection, post-labor fever, neonatal Apgar score, perineum rupture, episiotomy, mother’s satisfaction with the delivery process and the time of child staying in Neonatal Intensive Care Unit (NICU) were all recorded. 

α

## Results

A total of 200 mothers were studied as the two equal groups. Demographic characteristics of the mothers in the physiologic and intervention group were not statistically different. Among 100 control and 100 intervention cases, 22 patients underwent cesarean section. The number of cesareans in the facilitated group was more in comparison with the physiologic group. The type of childbirth (vaginal or cesarean) was found to be noticeably different in the two studied groups. The first stage of labor in physiologic group was observed to be shorter than the intervention group but this time difference was not statistically significant (p = 0.21). Although the second stage of the physiologic labor lasted longer as compared to the intervention group, this difference in the length of time was not considerable (p = 0.34). Moreover the duration difference of the third stage of the labor in the two groups was not statistically significant (p = 0.92). The comparison of the physiologic and facilitated born babies’ Apgar score showed no significant difference. Two of the facilitated born babies had an Apgar score below 7 and were admitted at NICU. The first stage of the labor in the two groups was different in terms of the maternal VAS (p = 0.001) whereas the second stage did not differ noticeably. 5% of all the physiologic labor patients showed abnormal vaginal bleeding which was remarkably lower compared with the intervention group (17%) as shown in Table 1. During the study, one case of the physiologic group and three cases of the facilitated group picked up the puerperal infection. The physiologic labor provided noticeably more gratification in the mothers compared with the facilitated labor (Table 1).

**Table 1:** The characteristics of patients in the two groups

		Non-pysiologic pregnancy group (n = 100)	Physiologic pregnancy group (n = 100)	p-value
**Age (year) (mean** **±** ** SD)**		**26.58** **±** ** 5.05**	**26.95** **±** ** 6.30**	**0.57**
**Gestational age by LMP (week) (mean** **±** ** SD)**		**38.71 ** **±** ** 1.15**	**38.70** **±** ** 0.91**	**0.95**
**Gestational age by sonography) (week) (mean** **±** ** SD)**		**38.62** **±** ** 0.65**	**38.36** **±** ** 4.08**	**0.53**
**Duration of delivery phase 1 (minute) (mean** **±** ** SD)**		**293.34 ** **±** ** 114.67**	**269.7** **±** ** 106.35**	**0.21**
**Duration of delivery phase II (minute) (mean** **±** ** SD)**		**35.02 ** **±** ** 14.07**	**37.33** **±** ** 17.69**	**0.34**
**Duration of delivery pahse III (minute) (mean ** **±** ** SD)**		**7.25 ** **±4.56**	**7.20** **±** ** 2.38**	**0.92**
**Apgar (mean** **±** ** SD)**	**First minute**	**8.83** **± ** ** 0.11**	**8.85 ** **±** ** 0.12**	**0.91**
	**Fifth minute**	**9.86 ** **±** ** 0.58**	**9.89** **± ** ** 0.75**	
**VAS (mean** **±** ** SD)**	**Phase I**	**7.81 ** **±** ** 0.62**	**6.90** **±** ** 0.88**	**0.001**
	**Phase II**	**9.89** **±** ** 0.34**	**9.81 ** **±** ** 0.46**	**0.21**
**Bleeding (%)**	**Abnormal**	**15**	**5**	**0.001**
	**Normal**	**72**	**89**	
**Perineal injury (%)**	**Normal**	**2**	**21**	**0.001**
	**Episiotomy**	**48**	**35**	
	**Laceration**	**32**	**39**	
**Satisfaction (%)**	**Low**	**24**	**6**	**0.001**
	**Moderate**	**57**	**25**	
	**Good**	**17**	**53**	
	**Excellent**	**1**	**16**	
**Infection (n)**		**3**	**1**	**0.33**

VAS= visual analogue scale

## Discussion

In the recent decades, a great number of the studies have explored the physiologic labor process. However, in Iran, investigating this process has just recently been initiated, and a few numbers of studies with the low sample size in one or two clinics have revealed the advantages and disadvantages of the physiologic labor. Based on a clinical trial performed in Tabriz-Iran, to compare the impact of the physiologic and facilitated labor on the neonatal status, the FHR of the born babies in the two methods varied significantly. The heart rate of the physiologic born babies was more in the normal range compared with the under-care ones. Also babies of the two groups were noticeably different in terms of the 5^th^ minute Apgar score, arterial blood pH, need for neonatal resuscitation and the time length of being admitted at neonatal unit (11). However, the same group did not explore the mother outcomes in the two delivery ways. On the contrary, this study showed not only no considerable difference in babies’ Apgar scores but it also studied the mother outcomes in both the physiologic and intervention labor. It has been reported that physiologic labor shortens the duration of the first stage of the delivery (12). However, this study showed no significant difference between the two groups in terms of the first stage of the labor duration. As contrast to the results of this study, Sadler et al. in 2000 published that the labor intervention such as the oxytocin induction, repeated vaginal examinations and amniotomy make the first stage of the process short (13). According to an Iranian study, the second stage of the physiologic labor was observed to be shorter compared with the control group (14) while the results of this study showed that the first and the third stage of the labor in the intervention group was longer than the physiologic group although this difference was not considerable. Fenton et al. found that the labor intervention reduces the vaginal bleeding (15). The episiotomy in the labor process is of the great importance. The proponents of this procedure believe that the episiotomy is essential, since it prevents up-grade rupture of perineum. On the other hand, due to the complications associated with the episiotomy such as fecal incontinence after the childbirth, opponents reject it. In this study mothers of the physiologic group had noticeably a higher grade of the laceration compared with the second group whereas Costa et al. in 2006 stated that the physiologic and facilitated groups were almost the same in terms of the laceration grade (16). Christians et al. conducted an investigation in 2009 with the aim of (1) comparing different types of the labor (2) finding the relation between the place of birth (home or hospital) and the satisfaction with childbirth in 4 groups of mothers in Belgium and the Netherlands. As a result, mothers were more content with childbirth at home than delivering at the hospital. Also in both countries the midwife’s support satisfied mothers more than the MD’s support (17). This study showed significant difference of VAS between the two studied groups. It was lower in the physiologic group compared with the intervention group. Therefore mothers were considerably more satisfied with the physiologic labor. Moreover, as reported by Liston et al. in 2008, the induced deliveries resulted in more neonatal complications in comparison with the spontaneous natural childbirth (18). According to an Iranian clinical trial carried out on 370 prim gravid mothers, the result of the physiologic and under routine care groups considerably varied in terms of FHR (shown to be more in the normal range in the physiologic cases), 5^th^ minute Apgar score, arterial blood pH, the need for the neonatal resuscitation and the time length of being admitted at the neonatal unit, while this study showed no significant difference between the two groups in terms of the Apgar average score.

The main limitation of this study was the relatively small sample size. Moreover only the patients referring to Arash hospital were recruited. Thus, further multicentre investigations with larger series are recommended to validate the findings reported here. 

## Conclusion

Generally, this study demonstrated that the rate of the cesarean section in the physiologic group was less than the intervention group. Furthermore, the duration of the first stage of the labor (without any significant difference), hemorrhage, episiotomy and the pain score were found to be lower in the physiologic group compared with the intervention group. Hence, mothers of the first group were more content with the labor process in comparison with the second group. 
